# Subjective Overload and Psychological Distress among Dentists during COVID-19

**DOI:** 10.3390/ijerph17145074

**Published:** 2020-07-14

**Authors:** Eitan Mijiritsky, Yaira Hamama-Raz, Feng Liu, Abhay N. Datarkar, Luca Mangani, Julian Caplan, Anna Shacham, Roni Kolerman, Ori Mijiritsky, Menachem Ben-Ezra, Maayan Shacham

**Affiliations:** 1 Department of Otolaryngology, Head and Neck and Maxillofacial Surgery, Tel-Aviv Sourasky Medical Center, Sackler Faculty of Medicine, Tel Aviv 6139001, Israel; 2 The Maurice and Gabriela Goldschleger School of Dental Medicine, Tel Aviv University, Tel Aviv 6997801, Israel; 3 School of Social Work, Ariel University, Ariel 40700, Israel; razizik@bezeqint.net (Y.H.-R.); menbe@ariel.ac.il (M.B.-E.); Drmaayanshacham@gmail.com (M.S.); 4 Department of Stomatology, Peking University, Beijing 100034, China; dentistliufeng@126.com; 5 Department of Oral & Maxillofacial Surgery, Govermental Dental College and Hospital Nagpur Maharashtra, Maharashtra 440003, India; abhaydatarkar@yahoo.com; 6 Department of Chemical and Technological Sciences, University of Roma “Tor Vergata”, Via Montpellier 1, 00133 Rome, Italy; manganiluca@yahoo.it; 7 Private Practice, Aviva Dentistry Ltd., St Albans AL1 3EN, Hertfordshire, UK; julian.caplan@btinternet.com; 8 Lev Hasharon Medical Center, Netanya 42100, Israel; anna.kupcha@gmail.com; 9 Department of Periodontology and Dental Implantology, The Maurice and Gabriela Goldschleger School of Dental Medicine, Tel Aviv University, Tel Aviv 6997801, Israel; kolerman@netvision.net.il; 10 Department of Psychology, Tel Aviv-Yafo Academic College, Tel Aviv 6818543, Israel; orimijiritsky2@gmail.com

**Keywords:** subjective overload, psychological distress, occupational dentistry, stress, psychosocial factors, COVID-19

## Abstract

Psychological distress during the COVID-19 pandemic is not solely limited to SARS-CoV-2 infection. It may also be related to social, cultural, and environmental factors, which may act as additional stressors. The aim of the current study was to explore the association between psychological distress and subjective overload among dentists in different countries, and whether it is associated with COVID-19-related factors. A cross-sectional survey was conducted among 1302 dentists from China, India, Israel, Italy, and the UK, who filled out demographics data, COVID-19-related factor questions, subjective overload, and psychological distress scales. Our findings showed that the positive association between subjective overload and psychological distress was different among countries, suggesting higher rate of intensity in Italy compared to China, India, and Israel (the UK was near significance with China and Israel). The interaction variable of the subjective overload × psychological distress was significantly associated with a particular country, with those individuals reporting fear of contracting COVID-19 from patients, fear of their families contracting COVID-19, and receiving enough professional knowledge regarding COVID-19. Given the above, dentists were found to have elevated levels of subjective overload and psychological distress, which differed among the countries, presumably due to certain background issues such as social, cultural, and environmental factors.

## 1. Introduction

Since late December 2019, the now-known severe acute respiratory syndrome coronavirus 2 (SARS-CoV-2) has spread worldwide, leading to an international public health issue known as coronavirus disease 2019 (COVID-19) [1,2]. The WHO declared it a pandemic event on 30th January 2020 [3].

In the face of the COVID-19 pandemic, medical staff worldwide are facing constant and high-magnitude stress during their daily work, which entails elevated risk of infection, frustration, exhaustion, social isolation, and being apart from their families [4]. Such stressful events during the COVID-19 pandemic may lead to increased risk of developing anxiety and stress disorders among medical staff, such as post-traumatic stress disorder (PTSD) [5]. Consequently, these mental health issues may negatively affect the decision-making ability of the medical staff, leading to less than optimal treatment for their patients as well as deteriorating their psychological well-being [4].

Psychological distress during the COVID-19 pandemic is not solely limited to SARS-CoV-2 infection. It may be also related to social, cultural, and environmental factors, which may act as additional stressors. Therefore, we sought to explore the relation between psychological distress and subjective overload among dental staff with regard to various countries.

The SARS-CoV-2, as a respiratory virus, may be transmitted through direct contact, indirect contact, through small droplets, or indirect or direct contact with saliva [6]. In dental practice, possible transmission routes include airborne spread (such as from aerosols formed during dental procedures), contact spread, and contaminated surface spread [7]. As dentists work in close proximity to patients’ mouths, they are exposed to their breathing, blood, and saliva, and as such are at an elevated risk. In line with this notion, Meng et al. [2] found that, despite the use of protective measures such as masks and gloves, several dental staff members in Wuhan were found to have been infected with COVID-19 as part of their work, along with some of their close relatives. Moreover, recent data suggest that not only patients with symptomatic COVID-19 act as a source of transmission, but also asymptomatic cases and those cases where the virus is still in its incubation phase. These act as additional stress factors for the treating medical staff [8,9,10]. These, in turn, may affect the dentists’ subjective overload.

Subjective overload is a psychological term, which in the case of dentists, may relate to their perceptions and understanding of their given circumstances. This is not limited to their dental practice, but rather involves other aspects of their everyday lives [11]. In addition, it relates to their coping mechanisms, and it may determine their level of job stress [12]. Given this, a recent study found among Israeli dental staff a positive link between subjective overload and psychological distress [13]. However, it might be that dentists in different countries will exhibit different levels of subjective overload due to their healthcare systems’ instructions, which in turn may affect their psychological distress.

Indeed, since the WHO declaration of COVID-19 pandemic, dental authorities worldwide have ordered dental clinics to treat only dental emergency cases (e.g., [14]). In addition, several studies (e.g., from China and Italy) have offered different recommended infection-control protocols for dental practices [2,7,15,16]. These recommendations demand a change in traditional dental infection-control measures and require different adjustments to be made by all involved sections, including dental health policy-makers and the dental staff. Thus, subjective overload might be increased, which in turn may cause additional psychological distress.

To sum up, as dentists in each country follow the guidelines provided by their dental authorities, and based on social, cultural, and environmental diversity, we aimed to evaluate the association between psychological distress and subjective overload among dentists in different countries, and whether it is associated with such diversity and with COVID-19-related factors.

## 2. Materials and Methods

### 2.1. Sampling and Procedure

We used an internet platform to conduct the survey after the study was approved by the institutional review board of the authors’ (M.S., Y.H-R., M.B.E.) university. The manuscript is in compliance with the STrengthening the Reporting of OBservational studies in Epidemiology (STROBE) statement. Each participant signed an electronic informed consent form. The participants were approached using social media such as Facebook, LinkedIn, WeChat, dedicated mailing lists from personal contacts of the authors’ addresses book (E.M., F.L., A.N.D., L.M., J.C., M.S.), and forums dedicated to dental professionals. A call to participate in the study was published once through social media, and, in accordance, dentists on mailing lists were contacted only once. No incentives were offered to the participants. Moreover, in order to prevent duplications, we used the SPSS code to find and remove identical cases on all variables in the sample.

During the period of 30 March to 12 April 2020, we collected data regarding dentists from China (*n* = 515), India (*n* = 470), Israel (*n* = 202), Italy (*n* = 88), and the UK (*n* = 27). A total of 1302 dentists responded to the survey. Basic demographics of each sample and the whole sample can be found in Table 1.

### 2.2. Measurements

Beyond basic demographic details, the following self-report questions were asked.

#### 2.2.1. COVID-19-Related Factors

Being in a risk group was measured by the question, “Are you defined as a high-risk population (suffering from chronic lung disease or moderate to severe asthma/chronic kidney disease and who are undergoing dialysis/liver disease/serious heart conditions/conditions that can cause a person to be immunocompromised, including cancer treatment/diabetes?”, coded as “0” for “No” and “1” for “Yes”.

Fear of contracting COVID-19 from patients was measured by the question, “Are you afraid that you will be infected with COVID-19 because of your profession?”, coded from “0” for “Not at all” to “4” for “Very afraid”.

Fear of family contracting COVID-19 was measured by the question, “Are you afraid that you will infect your family with COVID-19 due to your profession?”, coded from “0” for “Not at all” to “4” for “Very afraid”.

Receiving enough professional knowledge regarding COVID-19 was measured by the question, “Do you feel that you acquired sufficient knowledge (lectures, seminars, information leaflet, etc.) regarding maintaining a safe working environment since the COVID-19 outbreak?”, coded from “0” for “Not at all” to “4” for “Very much”.

#### 2.2.2. Psychological Factors

Subjective overload was measured using the Demands Scale—Short Version [12]. These six items looked at various aspects of personal stress: (1) “I cannot handle the contradicting demands required from me during my work”; (2) “The amount of work time available to me is insufficient”; (3) “My job poses demands without having the right equipment and resources”; (4) “I would never leave my work feeling that I have finished all my chores”; (5) “I am unable to perform my job to the best of my ability given the time allocated”; (6) “I am required to perform simple chores that prevent me from performing more sophisticated ones”, ranging from “1” for to “Not at all” to “5” for “Very much”. Cronbach’s alpha for the Demands Scale in the current study was 0.87.

#### 2.2.3. Outcome Variable

Psychological distress was measured using Kessler’s K6 [17], which included items on feeling nervous, hopeless, restless/fidgety, depressed, that everything was an effort, and worthless in the last 30 days. Scores ranged from 0 to 30, with 19 or higher indicating elevated psychological distress. Cronbach’s alpha for the Kessler’s K6 Scale in the current study was 0.86.

### 2.3. Statistical Analyses

The analytic plan had several stages: (1) We computed simple correlations between subjective overload and psychological distress for each country separately. (2) We compared the correlations between the countries using Fisher r-to-z transformation. (3) We used a post-hoc Scheffé adjustment to measure the differences in age, contracting COVID-19 from a patient, infecting the family with COVID-19, receiving enough information regarding COVID-19, subjective overload, psychological distress, and the new composed variable (subjective overload × psychological distress). In addition, we used Kruskal–Wallis tests for differences across the countries on the following variables: gender, marital status, and being in a risk group. (4) We created a new variable, namely subjective overload × psychological distress. This variable was the result of the multiplication of the two scales score (non-dichotomized). (5) We used analysis of covariance (ANCOVA) to measure the difference of the new variable (subjective overload × psychological distress), which served as the dependent variable, and the country served as the independent variable, while holding age, sex, marital status, background illness, fear of contracting COVID-19 from patients, fear of family contracting COVID-19, and receiving enough professional knowledge regarding COVID-19 as covariates. (6) We produced parameter estimates for the ANCOVA conducted in Step 5 in order to fit the model as a linear regression with regression parameter estimates and 95% confidence. (7) We produced an adjusted mean (SD) and 95% confidence interval for each country for the new variable (subjective overload × psychological distress). Finally, a multivariate analysis of covariance MANCOVA test similar to the ANCOVA, but with subjective overload and psychological distress as the joint bivariate dependent variables, was used. Data were analyzed using SPSS version 25 (IBM, Armonk, NY, USA).

## 3. Results

In this international study, Kruskal–Wallis tests and post-hoc analyses using the Scheffé post-hoc criterion for significance revealed significant differences among countries for age (F = 66.62, *p* < 0.0001), gender (Kruskal–Wallis H = 25.99, *p* < 0.001), marital status (Kruskal–Wallis H = 545.76, *p* < 0.001), being in a risk group (Kruskal–Wallis H = 58.29, *p* < 0.001), fear of contracting COVID-19 from patients (F = 8.53, *p* < 0.0001), fear of family contracting COVID-19 (F = 23.25, *p* < 0.0001), and feeling of having received enough professional knowledge regarding COVID-19 (F = 22.97, *p* < 0.0001), subjective overload (F = 9.09, *p* < 0.0001) and psychological distress (F = 2.49, *p* < 0.05). See Table 1 for further information.

In order to assess the correlations between subjective overload and psychological distress across the countries, Fisher Z-transformation analyses were used. The comparison of correlations between subjective overload and psychological distress across the countries was the highest among Italian dentists in comparison to the Chinese dentists (Z = −2.76, *p* < 0.01), Indian dentists (Z = −2.09, *p* < 0.05), and Israeli dentists (Z = −2.45, *p* < 0.05). See Table 2 for more information.

A one-way ANCOVA was conducted to compare the associations between the combined variable (subjective overload × psychological distress) whilst the country was the independent variable (F = 8.75; partial η^2^ = 0.026; *p* < 0.001). Among the covariates, the dependent dummy variable was significantly associated with those individuals reporting fear of contracting COVID-19 from patients (F = 76.22; partial η^2^ = 0.056; *p* < 0.001), fear of one’s family contracting COVID-19 (F = 25.02; partial η^2^ = 0.019; *p* < 0.001), and receiving enough professional knowledge regarding COVID-19 (F = 8.66; partial η^2^ = 0.007; *p* < 0.05) (see Table 3).

The means and 95% C.I. of the variables (subjective overload × psychological distress) varied between the countries. The lowest score was demonstrated in Israel (156.041; SE = 9.867; 95% CI 208.179–229.102). The highest score was demonstrated in the UK (244.396; SE = 20.610; 95% CI 203.964–284.828). See Table 4 for more details.

**Figure 1:**
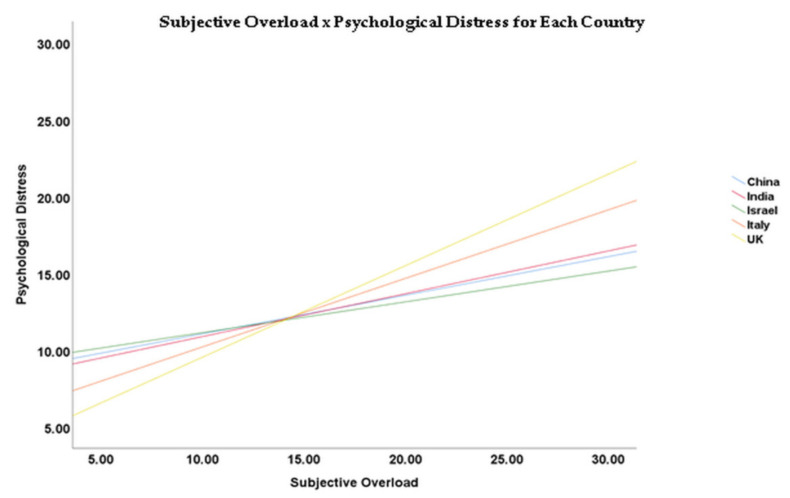
The association between subjective overload and psychological distress for each country.

The results of the parameter estimates for the ANCOVA presented in Table 3 revealed that marital status was negatively associated with the variable subjective overload × psychological distress (B = −18.853; SE = 7.155; t = −2.635; *p* = 0.009), as was having enough information regarding COVID-19 (B = −10.684; SE = 3.975; t = −2.700; *p* = 0.007). However, fear of being infected by a patient was positively associated with the variable subjective overload × psychological distress (B = 35.759; SE = 3.986; t = 8.972; *p* < 0.001), as was fear of infecting family (B = 18.084; SE = 4.016; t = 4.503; *p* < 0.001). See Table 5 for more details. In addition, please see Figure 1 for the different slopes across countries.

Finally, the results of the MANCOVA revealed that fear of contracting COVID-19 from a patient was associated with higher subjective overload (F = 30.506; *p* < 0.001; partial η^2^ = 0.023) and psychological distress (F = 66.828; *p* < 0.001; partial η^2^ = 0.049). The same was demonstrated for fear of infecting family with COVID-19, and this also was associated with higher subjective overload (F = 15.787; *p* < 0.001; partial η^2^ = 0.012) and psychological distress (F = 20.629; *p* < 0.001; partial η^2^ = 0.016). The opposite was found for having enough information on how to be protected from COVID-19, and this was negatively associated with subjective overload (F = 4.597; *p* = 0.032; partial η^2^ = 0.004) and psychological distress (F = 15.071; *p* < 0.001; partial η^2^ = 0.012). Finally, country was associated with subjective overload (F = 8.221; *p* < 0.001; partial η^2^ = 0.025) and psychological distress (F = 4.023; *p* = 0.003; partial η^2^ = 0.012). See Supplementary Table S1 (online supporting material).

## 4. Discussion

The current study focused on the association between subjective overload and psychological distress among dentists from five countries during the COVID-19 pandemic outbreak, and explored factors which were associated with this association. Our findings indicate that the positive association between subjective overload and psychological distress was different among countries, suggesting a higher association in Italy in comparison to China, India, and Israel. In addition, the association between subjective overload and psychological distress seemed to be highly correlated with certain COVID-19-related factors, namely: fear of contracting COVID-19 from patients, fear of one’s family contracting COVID-19, and receiving enough professional knowledge regarding COVID-19. Specifically, with regard to the higher association between subjective overload and psychological distress in Italian dentists in comparison to Chinese, Indian, and Israeli dental staff (the UK had comparable significance with China and Israel), this might be explained by the difference in the nature of job stress among dental staff with different cultural and national backgrounds [18]. In addition, culture by itself may affect the way an individual will perceive occupational stress [19].

Accordingly, the transactional theory of stress and coping (TTSC) [20] presents stress as the product of a transaction between a person (including multiple systems: cognitive, physiological, affective, psychological, neurological) and his or her complex environment. In line with this notion, Armocida et al. [21] argued that Italy faced a massive burden from the coronavirus disease 2019 (COVID-19) pandemic, as the Italian decentralization and fragmentation of health services seems to have restricted timely interventions and effectiveness. This was exacerbated by the fact that the healthcare systems’ capacity and financing were not flexible enough to take into account exceptional emergencies. Taking into account that many dentists in Italy are working in private settings, it might be that Italian dental staff experienced higher job stress in association with the above-mentioned conditions, which in turn caused higher psychological distress in comparison to their counterparts from the other countries. The rates of active COVID-19 cases and total death rates varied in each country over the time the survey was conducted. On 30 March, Israel had 4518 active cases and total of 20 death cases; Italy had 75,528 active cases and 11,591 deaths; China had 2161 active cases and 3305 deaths; India had 1117 active cases and 32 deaths; and the UK had 22,141 active cases and 2042 deaths. On 12 April, Israel had 9413 active cases and 103 deaths; Italy had 102,253 active cases and 19,468 deaths; China had 1156 active cases and 3341 deaths; India had 7794 active cases and 331 deaths; and the UK had 84,279 active cases and 12,285 deaths [22]. Due to epidemiological limitations, it may be problematic to compare trends between countries, as recently suggested [23], since there were different infection and death rates among the countries and different definitions for “coronavirus death”. Nonetheless, these rates may exert different effects on subjective overload and psychological distress as experienced by different individuals from different cultures and countries.

With regard to the associations between subjective overload and psychological distress with fear of contracting COVID-19 from patients, fear of one’s family contracting COVID-19, and receiving enough professional knowledge regarding COVID-19, this may be supported by evidence gathered during the SARS epidemic. Levels of fear of contracting SARS increased job stress related to SARS, and have been found to be associated with post-traumatic symptoms among healthcare providers [24,25]. Moreover, according to Meng et al. [2], due to the unique characteristics of dental procedures, where a large number of droplets and aerosols could be generated, the standard protective measures in daily clinical work are not effective enough to prevent the spread of COVID-19. This is especially important as when patients are in the incubation period, are unaware that they are infected, or choose to conceal their infection. Thus, the fear of the dental staff contracting COVID-19 from patients, as well as fear of their families contracting COVID-19, may be reasonable in such conditions. Another explanation may stem from the uncertainty in illness theory [26,27], which argues that situations with uncertainty include some lack of information, especially if the information that is required for decision-making is unknown, vague, unpredictable, or ambiguous. As guidelines regarding possible SARS-CoV-2 transmission routes and recommended infection-control measures among healthcare providers continue to emerge, it is not surprising that providing professional knowledge regarding COVID-19 was found to be related to the association between subjective overload and psychological distress.

Several limitations of the current study should be noted. The cross-sectional design of the study did not allow any inferences regarding causal relationships. Further, the study’s findings relied on self-report measures and were collected over a relatively short period of time. The study was subject to selection bias and sampling error, as participants were approached using social media, dedicated mailing lists, and forums. Data regarding how many dentists were actually engaging in dentistry during the COVID-19 pandemic were not probed in our study. Data regarding the age of each country’s dentists compared to the age of the participants served as an additional limitation. Thus, a longitudinal design would provide more reliable indications on the stability of the association between psychological distress and subjective overload among dentists in different countries, and of the COVID-19-related factors which were found to be linked to this association. Additionally, as the survey was conducted at times of a worldwide pandemic event, it was difficult to assess whether culture solely affected subjective overload and psychological distress, or whether it was the pandemic itself which led to their elevated levels. The dynamics of the COVID-19 pandemic in each country may be differently correlated with subjective overload and physiological distress, and, as this was beyond the scope of our study, we did not address it.

Furthermore, it may be that respondents hold views that are not representative of all dentists in each country. In addition, as recruitment was done by public posting in some cases, we could not estimate the response rate by country. Finally, some countries had lower rates of survey participation, which may have been due to a lack of interest by dentists, possibly due to the severity of the disease in these countries when the survey was conducted, or/and their prioritized their efforts in better understanding the COVID-19 crisis and its implications for the dental profession. Nevertheless, the study sample was of a large enough size to provide statistically significant results.

## 5. Conclusions

This study provides insights into the mental health of dentists in five countries during a period the COVID-19 epidemic outbreak. Our findings indicated elevated levels of subjective overload and psychological distress among dentists, which differed between the countries. As background issues such as social, cultural, and environmental factors presumably play a role in such differences, and in order to minimize the long-term effects of such psychological distress among dentists, future implications, such as providing mental health workshops for dentists, should be addressed. In addition, introduction of essential knowledge and recommended management protocols for dental practitioners is warranted, in order to reduce personal and professional fears of contagion. Clinical dental implications from this study that could positively affect the association between subjective overload and physiological distress may include providing dental staff with information/guidelines regarding implementation of innovative infection-control methods including personal protective equipment (PPE), which might provide a sense of safety, and in turn might reduce psychological distress and subjective overload.

## Figures and Tables

**Table 1:** Basic demographic and country comparison based on the study variables, using Kruskal–Wallis and post-hoc Scheffé tests.

	International Sample (*n* = 1302)						
Factors	China (*n* = 514)	India (*n* = 470)	Israel (*n* = 202)	Italy (*n* = 88)	United Kingdom (*n* = 27)	Tests	Post-Hoc Scheffé
Age, years (M/SD)	38.7 (8.3)	34.9 (9.4)	47.0 (11.4)	44.8 (12.5)	44.7 (9.6)	F = 66.61 ***	1 < 2, 1 < 2, 1 < 3, 1 < 4, 1 < 5, 2 < 3, 2 < 4, 2 < 5
Sex, male % (*n*)	55.4 (285)	54.3 (255)	69.8 (141)	73.9 (65)	(70.4) 19	Kruskal–Wallis H = 25.99 ***	
Committed relationship, % (*n*)	97.1 (499)	64.7 (304)	80.2 (162)	80.7 (71)	85.2 (23)	Kruskal–Wallis H = 545.76 ***	
Background Illness, % (*n*)	5.8 (30)	7.7 (36)	23.3 (47)	4.5 (4)	11.1 (3)	Kruskal–Wallis H = 58.29 ***	
Contract COVID-19, (M/SD)	2.5 (.9)	2.4 (.9)	2.7 (.8)	2.5 (.9)	3.1 (.8)	F = 8.53 ***	1 < 3, 1 < 5, 2 < 3, 2 < 5, 4 < 5
Infect family COVID-19, (M/SD)	2.3 (.9)	2.5 (.9)	3.0 (.9)	2.8 (.9)	3.1 (.9)	F = 23.25 ***	1 < 3, 1 < 4, 1 < 5, 2 < 3, 2 < 5,
Received information regarding COVID-19, (M/SD)	3.0 (.6)	2.7 (.7)	2.8 (.9)	2.8 (.9)	2.0 (.9)	F = 22.97 ***	1 > 2, 1 > 3, 1 > 5, 2 > 5, 3 > 5, 4 > 5
Subjective overload, (M/SD)	16.1 (4.6)	14.8 (4.8)	14.4 (5.8)	15.7 (5.7)	18.6 (4.8)	F = 9.09 ***	1 > 2, 1 > 3, 2 < 5, 3 < 5,
Psychological distress, (M/SD)	12.6 (4.7)	12.2 (4.2)	12.0 (4.6)	12.7 (4.8)	14.6 (5.0)	F = 2.49 *	
Subjective overload × psychological distress	208.5 (114.7)	187.32 (103.8)	180.45 (115.9)	213.92 (143.3)	284.0 (148.0)	F = 7.663 ***	1 < 5, 2 < 5, 3 < 5

* *p* ≤ 0.05; *** *p* ≤ 0.001.

**Table 2:** Correlations between subjective overload and psychological distress across the countries and correlation comparison using Fisher Z-transformation (*n =* 1302).

	China	India	Israel	Italy	UK
Correlation between subjective overload and psychological distress	0.245	0.316	0.251	0.518	0.571
Country comparison	China # Italy: Z = −2.76; *p* < 0.01	India # Italy: Z = −2.09; *p* < 0.05	Israel # Italy: Z = −2.45; *p* < 0.05	All except UK.	Near significance with China and Israel.
*n*	515	470	202	88	27

**Table 3:** ANCOVA results including effect size for subjective overload × psychological distress (*n* = 1302) (Significance ** *p* ≤ 0.01; *** *p* ≤ 0.001).

	Type III Sum of Squares	df	F	Partial η^2^
Country	385,071.69 ***	4	8.75	0.026
Age	67.66	1	0.01	0.000
Sex	21,826.16	1	1.98	0.002
Committed relationship	9118.02	1	0.83	0.001
Background illness	2090.83	1	0.19	0.000
Contract COVID-19	838,902.81 ***	1	76.22	0.056
Infect family COVID-19	275,410.73 ***	1	25.02	0.019
Received information regarding COVID-19	95,282.55 **	1	8.66	0.007

**Table 4:** Mean and 95% confidence intervals for the dependent variable (subjective overload × psychological distress) appearing in the model.

Country	Mean	Std. Error	95% Confidence Interval	
			Lower Bound	Upper Bound
China	218.641	5.333	208.179	229.102
India	189.833	5.068	179.891	199.775
Israel	156.041	9.867	136.684	175.398
Italy	209.444	11.420	187.040	231.847
UK	244.396	20.610	203.964	284.828

**Table 5:** Parameter estimates for the ANCOVA results (Table 3) in order to fit the model as a linear regression with regression parameter estimates and 95% confidence interval (significance ** *p* ≤ 0.01; *** *p* ≤ 0.001).

Parameter	B	Std. Error	t	95% Confidence Interval	
				Lower Bound	Upper Bound
Intercept	144.251 ***	25.089	5.750	95.032	193.470
Age	−0.145	0.303	−0.477	−0.739	0.450
Sex	−7.591	6.232	−1.218	−19.816	4.634
Marital status	−18.853 **	7.155	−2.635	−32.889	−4.816
Country	−4.322	3.440	−1.256	−11.071	2.427
Risk group	−1.602	10.533	−0.152	−22.265	19.061
Fear of being infected by patient	35.759 ***	3.986	8.972	27.940	43.579
Fear of infecting family	18.084 ***	4.016	4.503	10.205	25.964
Having enough information regarding COVID-19	−10.684 **	3.957	−2.700	−18.446	−2.922

## Supplementary Materials

The following are available online at https://www.mdpi.com/1660-4601/17/14/5074/s1, Table S1: MANCOVA results including effect size for subjective overload X psychological distress (n= 1302).
