# Supplementary material for: Subjective Overload and Psychological Distress among Dentists during COVID-19
Source: Int J Environ Res Public Health. 2020 Jul 14;17(14):5074. doi: 10.3390/ijerph17145074 (PMC7399825; doi:10.3390/ijerph17145074)
Supplement: Supplementary file 1 [file ijerph-17-05074-s001.pdf]

Online Supporting Martial

Table S1. MANCOVA results including effect size for subjective overload X psychological distress (n= 1302)

Source	DV	Type III Sum of Squares	Mean Square	F	Sig.	Partial Eta Squared
Age	SO	53.019	53.019	2.337	.127	.002
	K6	37.253	37.253	2.101	.147	.002
Sex	SO	121.647	121.647	5.362	.021	.004
	K6	1.456	1.456	.082	.775	.000
Marital Status	SO	.405	.405	.018	.894	.000
	K6	23.543	23.543	1.328	.249	.001
High Risk for COVID-19	SO	5.064	5.064	.223	.637	.000
	K6	6.451	6.451	.364	.547	.000
Fear of Contracting COVID-19 from Patient	SO	692.031	692.031	30.506	.000	.023
	K6	1184.940	1184.940	66.828	.000	.049
Fear of Infecting the Family with COVID-19	SO	358.131	358.131	15.787	.000	.012
	K6	365.772	365.772	20.629	.000	.016
Information how to Protect Against COVID-19	SO	104.279	104.279	4.597	.032	.004
	K6	267.234	267.234	15.071	.000	.012
Country	SO	745.943	186.486	8.221	.000	.025
	K6	285.305	71.326	4.023	.003	.012

DV = Dependent Variable; So = Subjective Overload; K6 = Psychological Distress
